# Human Medial Frontal Cortex Mediates Unconscious Inhibition of Voluntary Action

**DOI:** 10.1016/j.neuron.2007.05.016

**Published:** 2007-06-07

**Authors:** Petroc Sumner, Parashkev Nachev, Peter Morris, Andrew M. Peters, Stephen R. Jackson, Christopher Kennard, Masud Husain

**Affiliations:** 1 School of Psychology, Cardiff University, Tower Building, Park Place, Cardiff CF10 3AT, UK; 2 Division of Neuroscience, Faculty of Medicine, Imperial College London, St Dunstan's Road, London W6 8RP, UK; 3 Magnetic Resonance Centre, School of Physics and Astronomy, University of Nottingham, University Park, Nottingham NG7 2RD, UK; 4 School of Psychology, University of Nottingham, University Park, Nottingham NG7 2RD, UK; 5 Institute of Neurology and Institute of Cognitive Neuroscience, University College London, 17 Queen Square, London WC1N 3AR, UK

**Keywords:** SYSNEURO

## Abstract

Within the medial frontal cortex, the supplementary eye field (SEF), supplementary motor area (SMA), and pre-SMA have been implicated in the control of voluntary action, especially during motor sequences or tasks involving rapid choices between competing response plans. However, the precise roles of these areas remain controversial. Here, we study two extremely rare patients with microlesions of the SEF and SMA to demonstrate that these areas are critically involved in unconscious and *involuntary* motor control. We employed masked-prime stimuli that evoked automatic inhibition in healthy people and control patients with lateral premotor or pre-SMA damage. In contrast, our SEF/SMA patients showed a complete reversal of the normal inhibitory effect—ocular or manual—corresponding to the functional subregion lesioned. These findings imply that the SEF and SMA mediate *automatic* effector-specific suppression of motor plans. This automatic mechanism may contribute to the participation of these areas in the voluntary control of action.

## Introduction

Regions within the dorsal medial frontal cortex are considered to play a key role in the voluntary control of action (Passingham, 1993). In particular, the supplementary motor area (SMA), supplementary eye field (SEF), and the pre-SMA have repeatedly been implicated in actions that are “self-initiated” or driven by “internal goals” (e.g., Coe et al. [2002], Deiber et al. [1999], Isoda and Hikosaka [2007], Lau et al. [2004], Nachev et al. [2005], Passingham [1993], Romo and Schultz [1987], Tanji and Shima [1994]). For example, lesions or reversible inactivation of SMA and pre-SMA in macaques have caused deficits in simple movements and motor sequences when they were self initiated, but not when guided by sensory cues (Chen et al., 1995; Nakamura et al., 1999; Shima and Tanji, 1998; Thaler et al., 1995). In humans, lesions restricted to these areas are rare, but studies of patient JR, who has a highly unusual focal lesion of SEF, have revealed deficits in voluntary eye movement control, such as the ability to switch from a previous saccade plan or between prosaccades toward a stimulus and antisaccades away from it (Husain et al., 2003; Parton et al., 2007).

Although there are clear distinctions between voluntary and reflexive actions, at some level all behavior must be built on a variety of automatic mechanisms, since there can be no homunculus to steer actions by specifying volitional goals. To explain the contribution of the medial frontal cortex to voluntary behavior, we seek to identify automatic processes upon which voluntary actions are built.

Behavior must arise through associations between particular contexts—including internal brain states—and particular actions and through the interplay and competition between these “condition-action associations” (Nachev et al., 2007; Wise and Murray, 2000). Thus all actions—including “self-initiated” or “internally generated” ones—depend upon context. But the defining criterion for voluntary behavior is that the stimulus environment does not inevitably specify one particular movement. In other words, there is *choice*, because more than one action contingency may be associated with the current set of conditions. But with choice there is always the potential for activation of unwanted movement plans—and the need to suppress such activity.

It is now clear that well established condition-action associations may cause *automatic* subconscious motor activation (or “priming”), even when the participant has no intention of making the associated movement. For example, simply viewing objects can prime manual responses that might be used to grasp such an object (Tucker and Ellis, 2004). Importantly, such activations occur in the SMA (Grezes and Decety, 2002). Motor activation can even be elicited by established condition-action associations without the stimuli themselves being consciously perceived—as in the case of masked-priming (e.g., Leuthold and Kopp [1998], Neumann and Klotz [1994]). Thus an inevitable feature of choice, the defining criterion of voluntary behavior, is the potential for automatic coactivation of action plans (Nachev et al., 2007).

If flexible behavior is to be possible though, such automatic motor activation must be inhibited so that the most strongly established actions are not inevitably executed. Moreover, this inhibitory mechanism can potentially be understood without invoking higher-order volitional goals. It may simply suppress any response activation triggered by changes in the sensory environment which would otherwise interfere with ongoing motor plans. Such automatic inhibition can be measured using a recently developed masked-prime task, outlined below (e.g., Eimer and Schlaghecken [2003], Schlaghecken et al. [2006]). By rapidly halting partial activation of strongly established condition-action associations and maintaining a level playing field on which alternative actions can occur, *automatic* mechanisms could thus contribute to flexible voluntary behavior. The aim of our study is to determine whether one of the mechanisms through which the SMA/SEF may contribute to voluntary control is automatic inhibition of unwanted motor plans (activated unconsciously by well-established condition-action associations).

**Figure 1:**
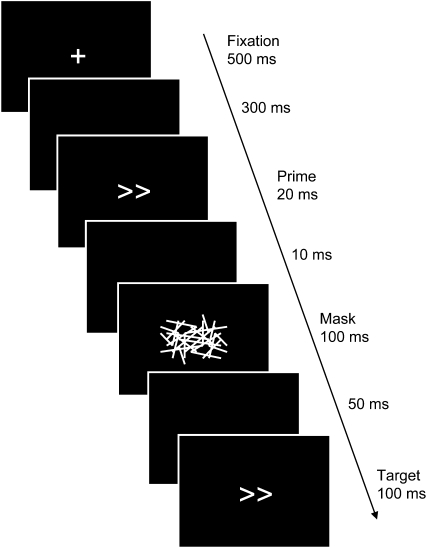
Illustration of the Stimulus Sequence in the Masked-Prime Task The participants responded to the target arrows, which could point left or right, by pressing left or right buttons. The prime, which could also point left or right, was rendered invisible by the mask, so that participants saw only the fixation cross, the mask, and the target. If the prime was the same as the target, as depicted, it is a “compatible” trial. If the prime points in the opposite direction, it is an “incompatible” trial. Note that following previous convention (e.g., Eimer and Schlaghecken [2003]), the trial's SOA is measured from the start of the mask until the start of the target: in this case 150 ms.

In the masked-prime task, which we employed here, participants respond to stimuli associated with left or right responses. These targets are preceded by brief primes rendered invisible by a backward mask (Figure 1). If the delay between prime and target is shorter than 100 ms, there is facilitatory priming, such that responses are faster when both prime and target indicate the same response (compatible), compared to when prime and target produce competition by indicating opposite responses (incompatible). However, at longer prime-target delays, a negative compatibility effect (NCE) is produced, such that now responses are actually *slower* for compatible primes than for incompatible primes (e.g., Eimer and Schlaghecken [2003]; Schlaghecken et al. [2006]). As long as potential prime-mask perceptual interactions are controlled (Lleras and Enns, 2004; Sumner, 2007b; Verleger et al., 2004), the NCE can be taken to index an automatic inhibitory mechanism that suppresses the subthreshold motor activation evoked by the prime, stopping it from interfering with an alternative response to the target (Jaskowski and Przekoracka-Krawczyk, 2005; Schlaghecken and Eimer, 2006; Sumner, 2007a; see Supplemental Data available with this article online for further details).

Note that there is no voluntary or conscious effort to suppress responses to the prime, because the prime is not perceived (Eimer and Schlaghecken, 2003; Sumner et al., 2006). Therefore, unlike previous tasks employed to study the functions of SMA and SEF, this task permits a distinction between higher-order executive mechanisms that represent volitional goals and lower-level fundamental mechanisms that automatically keep response alternatives in check. On occasions, such automatic mechanisms might appear maladaptive, suppressing actions that are subsequently required. But the slight delay this causes may be a small cost relative to risking the execution of an inappropriate action. Moreover, as outlined above, the inhibition does not occur immediately, allowing for facilitation by the primes as long as the same action is cued by the target at around the same time.

Critically, this automatic inhibition is effector specific, rather than operating at a perceptual or more general processing level (Eimer and Schlaghecken, 2003). For example, primes associated with left or right foot movements do not cause inhibition of manual responses (Eimer et al., 2002). Likewise, many neurons in the SMA and SEF have effector-specific activity, for hand and eye movements, respectively (Chainay et al., 2004; Fujii et al., 2002; Luppino et al., 1991). Thus, we hypothesize that some of the activity measured in this region during volitional behavior might be related to the effector-specific automatic inhibition of subconsciously activated condition-action associations, as measured by the masked-prime task.

It is challenging, however, to establish a causal relationship between a brain area and a specific function. Documenting brain activity, using fMRI for example, might support such an argument, but in order to demonstrate that an area is essential for a function, it is necessary to study the effects of interfering with that region. Unfortunately, lesions in humans are usually large, spanning several different brain areas, therefore making it difficult to come to any definitive conclusion. Here, we overcome these limitations by studying patients with rare and serendipitously located lesions.

**Figure 2:**
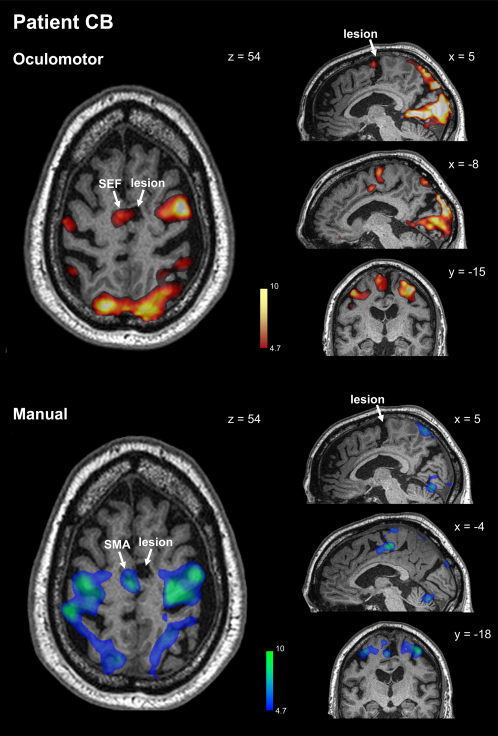
Functional Localization of CB's Lesion Statistical contrasts (thresholded at p < 0.001 uncorrected) for oculomotor activity (upper panels) or manual activity (lower panels) are superimposed on a T1-weighted anatomical scan. Oculomotor activity within the caudal SFG constitutes the functionally defined SEF and appears in the left hemisphere directly opposite the lesion in the right hemisphere. Likewise, manual activity within the caudal SFG constitutes the functionally defined SMA and appears opposite and caudal to the lesion. See text for more details. See Supplemental Data for clinical information and imaging methodology.

**Figure 3:**
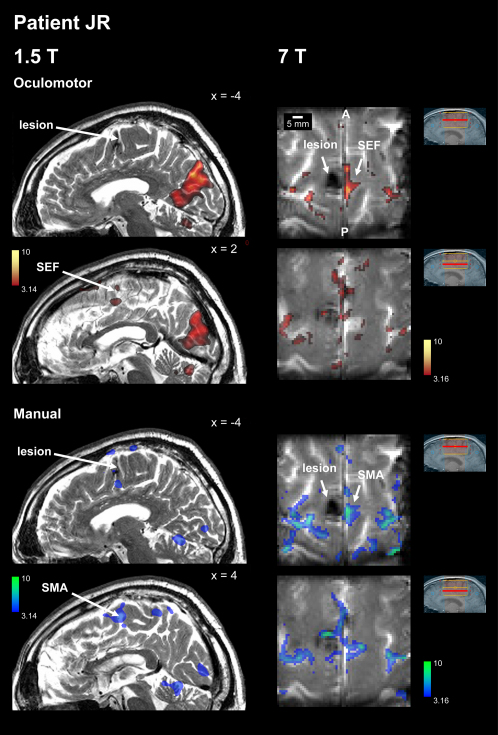
Functional Localization of JR's Lesion Functional images for oculomotor activity (upper panels) or manual activity (lower panels) were acquired at 1.5T (left panels) and at 7T (right panels). For 1.5T, statistical contrasts (thresholded at p < 0.001 uncorrected) are superimposed on a T2-weighted sagittal image acquired with resolution 1.6 × 0.575 × 0.575 mm. The high-resolution 7T functional maps (1 × 1 × 3 mm) are superimposed on the mean echoplanar image. The scale bars indicate the very small size of the lesion (note also that there is likely to have been signal dropout around the true lesion due to hemosiderin deposition). There is clear contralesional oculomotor activity precisely opposite the lesion, indicating that the ipsilesional SEF is damaged. The extent of damage to the SMA is less clear but is likely to be less than to the SEF, given the rostral position and small size of the lesion. See text for more details. See Supplemental Data for clinical information and imaging methodology.

**Figure 4:**
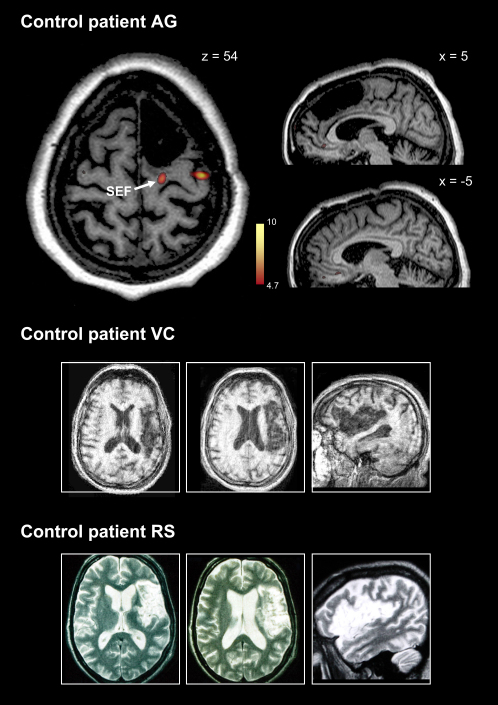
Control Patients' Lesions Functional imaging for oculomotor activity in patient AG (left panel) demonstrates SEF activation in the lesioned hemisphere caudal to the lesion. Thus pre-SMA is damaged, but not SEF and SMA. Sagittal structural images to either side of midline (right panels) reveal the extent of surgical resection in AG. Patient VC and patient RS have extensive strokes involving lateral frontal cortex, including ventral and dorsal premotor regions, but no involvement of medial frontal cortex. See Supplemental Data for clinical information and imaging methodology.

The first case, CB, has a focal lesion involving the SEF and SMA, as demonstrated using structural and functional imaging (Figure 2; see Supplemental Data for clinical details). The second patient, JR, has an even smaller lesion affecting the paracentral sulcus. New functional imaging performed for the first time at 7T in a human with a brain lesion indicates that the SEF is damaged (Figure 3), consistent with previous findings demonstrating deficits for eye—but not hand—movements in this patient (Husain et al., 2003). CB's and JR's lesions are by far the smallest ever studied in the medial frontal lobe and are on a par with the functional granularity of cortical areas revealed by fMRI or neurophysiology. These patients present a precious opportunity to study the specific contribution of the SMA and SEF to the control of action. The third medial frontal patient, AG, has a much larger lesion, which encompasses the pre-SMA (Figure 4). She also shows deficits in the voluntary control of movement, encountering difficulty in switching from one limb movement plan to another (Nachev et al., 2007). Remarkably, the caudal border of her lesion lies in the same position as the rostral borders of the other two patient's lesions, making AG's lesion an excellent control, allowing the specific damage in patients CB and JR to be compared to her more extensive damage involving the pre-SMA. Finally, we also studied two cases (RS and VC) who have very large frontal lesions involving lateral premotor areas (Figure 4). These patients act as further controls for comparing the consequences of extensive premotor damage to the specific damage to SMA and SEF in patients CB and JR.

If parts of the supplementary motor regions mediate automatic inhibition, as we hypothesize, we would expect them to do so in an effector-specific manner, with the SMA responsible for inhibition of manual movements and SEF responsible for inhibition of eye movements. Thus, while in CB we would expect disrupted inhibition for both saccades and manual responses, in JR we might predict a dissociation between manual and saccadic responses. The results demonstrate precisely this pattern, providing strong evidence that these regions are causally involved in automatic response inhibition in an effector-specific manner. By contrast, patients AG, RS, and VC displayed inhibition just like healthy age-matched controls, despite having lesions many times larger than those in CB and JR. These findings support the proposal for a dissociation between the mechanisms of control undertaken by SMA or SEF and those undertaken by other frontal areas such as the pre-SMA, which we consider in more detail below (see Discussion).

## Results

### Anatomical and Functional Imaging

The SMA is generally defined as the area of medial frontal cortex in the superior frontal gyrus lying dorsal to the cingulate sulcus, rostral to the primary motor cortex and caudal to the VCA line (Picard and Strick, 1996, 2001), and can be distinguished from the pre-SMA (rostral to the VCA line) by its pattern of connectivity (Johansen-Berg et al., 2004). The anatomical landmark of the SEF is generally taken to be the paracentral sulcus (Grosbras et al., 1999). However, between individuals there is considerable rostrocaudal variation in these functional areas, as shown by a meta-analysis of human imaging studies (Mayka et al., 2006). Consequently, in order to specify the lesion locations, we *functionally* localized the SEF and SMA in our medial frontal patients as well as ten healthy volunteers using simple tasks of voluntary eye movements or hand movements, interleaved with rest, during functional MRI (see Supplemental Data for details).

**Figure 5:**
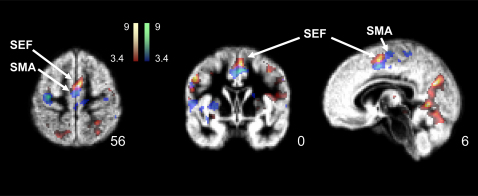
Functional Localization of SEF and SMA in Healthy Participants Oculomotor activity (red-yellow) occurred reliably just rostral to manual activity (blue-green) in the SFG, and activity was confluent across the medial wall of the SFG. Group data (n = 10) are shown; see Supplemental Data for individual data and imaging methods.

In the healthy participants, we confirmed that the peak oculomotor activity representing the SEF was reliably rostral to the peak manual activity representing the SMA (see Figure 5 and Supplemental Data), and that the location of either the SEF or SMA in one hemisphere is an excellent guide to the location of its homolog in the other, consistent with previous reports (e.g., Grosbras et al. [1999], Picard and Strick [1996]). In our patients, therefore, the location of the SEF and SMA in the *intact* hemisphere can be used as a reasonable guide to the location of these areas on the lesioned side. This is important because the presence of BOLD signal around the lesion itself may be due to the effects of task-correlated head movement at the lesion boundary, which may not be completely eliminated by including head realignment parameters in the design matrix (see Supplemental Data). Conversely, the absence of signal near lesions may be due to signal drop-out caused by hemosiderin deposition within damaged tissue which might nonetheless be functioning normally.

In patient CB, comparison of the eye-movement blocks with rest blocks (both coregistered with his anatomical image) revealed oculomotor activity in the left caudal superior frontal gyrus (SFG) in the area expected for the intact left SEF (Figure 2, top panels). Axial, sagittal, and coronal views all confirm that CB's lesion is located precisely opposite the functionally defined left SEF, indicating that the right SEF is damaged. As expected, oculomotor activity was also present in the visual cortex, parietal cortex, and frontal eye fields. Parallel analysis of the finger-movement blocks revealed activity bilaterally in the parietal cortex, motor, and premotor areas, as well as in the left caudal SFG, in the area expected for the intact left SMA (Figure 2, lower panels). This activity is opposite the lesion and also extends caudally, consistent with the results from healthy participants. From the anatomical scan, we computed the lesion volume to be 3.55 cm^3^ (which includes the sulcal area within the lesion and is thus an overestimate of cortical volume damaged). By comparison, from the Human Motor Area Template compiled by Mayka et al. (2006), we calculated the approximate average size of the normal supplementary motor complex (which includes the entire somatotopic representation in SMA and SEF) to be 9.8 cm^3^ in each hemisphere.

Patient JR's lesion was not demonstrated well using a standard T1-weighted sequence, but the T2-weighted anatomical image revealed a very small lesion (volume = 0.31 cm^3^) in the left caudal SFG at the paracentral sulcus, considered by some authors to be an anatomical landmark for the SEF (Grosbras et al., 1999). Functional localizers for SEF and SMA found oculomotor and manual activity in the intact right SFG opposite the lesion (Figure 3, left panels). The functionally defined SMA in the intact hemisphere (as indexed by hand movement-related activity) appeared to extend caudally to the lesion, while the SEF did not, consistent with previous reports that JR has a volitional deficit in the oculomotor domain, but not for manual actions (Husain et al., 2003; Parton et al., 2007).

We obtained further functional images with 7T fMRI (Figure 3, right panels), the first such scans on a patient with a focal lesion. These confirmed oculomotor activity in the region of the right SEF exactly opposite the lesion. Thus, despite the small size of the lesion revealed by these images, it seems clear that the left SEF is damaged. The situation for the left SMA is less immediately clear on these images. There was manual movement-related activity opposite the lesion, corresponding to right SMA, as well as some apparent manual activity in the lesioned hemisphere in the region expected for the left SMA, but note, as above, that we do not wish to draw conclusions from presence or absence of activity around the borders of lesions. However, given that the lesion is very small and aligned with the rostral edge of the SEF (as defined by contralesional oculomotor activity) and that the SMA hand representation is caudal to the SEF (as confirmed in healthy participants above) much of JR's SMA must be spared. Note also that the lesion may appear larger than it really is because of greater susceptibility to signal dropout from hemosiderin at 7T. Taken together, the 1.5T and 7T imaging for JR suggest that his lesion has damaged the left SEF and may involve part of the left SMA, but far less than in CB. This interpretation is consistent with previously reported behavioral eye-hand dissociations in JR (Husain et al., 2003; Parton et al., 2007).

For control patient AG, anatomical imaging demonstrates a much larger lesion of right medial frontal cortex, including rostral SFG. The oculomotor functional localizer revealed activity corresponding to the right SEF just caudal to the lesion in the right hemisphere (Figure 4). Because the SEF lies at the rostral boundary of the supplementary motor complex (Yamamoto et al., 2004), it represents an important landmark, marking the boundary with the pre-SMA. Thus, AG's lesion involves the pre-SMA (as well as part of the cingulate cortex), but not the supplementary motor complex. We were unable to perform a second scan on this patient to localize the SMA hand area definitively as she was unkeen to participate in further imaging studies.

We also studied two control patients, RS and VC, who have *lateral* frontal lesions which spare the medial frontal cortex but involve lateral premotor cortex (Figure 4). The lesions in both patients involved the inferior frontal gyrus, inferior frontal sulcus and middle frontal gyrus, as well as the underlying white matter. Since the inferior frontal sulcus is a guide to the boundary between dorsal premotor cortex (PMd) and ventral premotor cortex (PMv) (for a discussion see Mayka et al. [2006]), their brain damage involves both PMv and PMd, while sparing the medial wall of the superior frontal gyrus.

### Automatic Inhibition of Manual Responses

We used a masked-prime task with manual responses, based on the paradigm developed by Eimer and Schlaghecken (e.g., Eimer and Schlaghecken [2003], Schlaghecken et al. [2006]). Participants pressed a left or right button as fast as possible in response to arrows that pointed left or right (see Figure 1). Before each target arrow, there occurred a prime arrow (20 ms) followed by a mask of random lines (100 ms), which rendered the primes nondiscriminable: No participant scored above chance when asked to discriminate the prime arrows (mean 52%), and all claimed not to see them. We did not employ masks of overlapping arrows, which have been criticized for interacting with the prime to produce confounding priming effects (Lleras and Enns, 2004; Sumner, 2007a; Verleger et al., 2004). See Experimental Procedures and Supplemental Data for more details.

**Figure 6:**
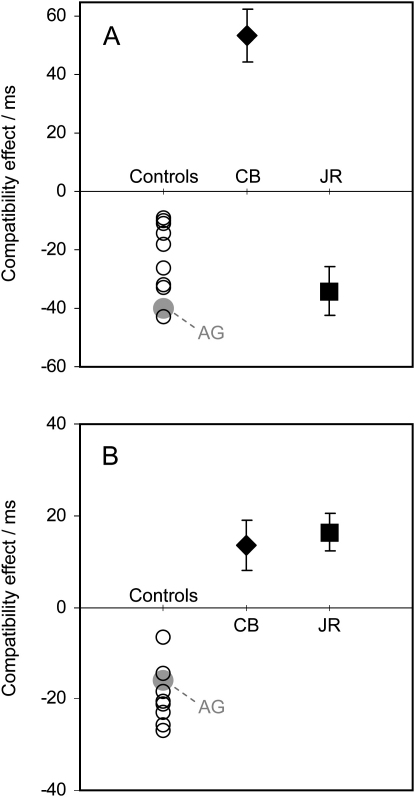
Results for Manual and Saccadic Responses The compatibility effect is the difference between response time on incompatible and compatible trials (and error bars are the standard error of this difference). For all control subjects, there was a negative compatibility effect—i.e., responses were longer on compatible trials—for both manual (A) and saccadic (B) responses, indexing inhibition (open circles for neurologically healthy controls; gray circle for AG, the lesion control patient). CB, however, showed a strong positive compatibility effect, indicating that the inhibitory process is disrupted for both manual and saccade responses. JR, whose smaller lesion appears to largely spare SMA, showed a normal negative effect for manual responses (A) but a positive effect for saccadic responses (B). See text for more details.

The primes were randomly either compatible (the same as the target arrow), or incompatible (opposite to the target arrow). At short prime-target intervals, primes produce facilitation, but for the longer intervals used in this study, there is expected to be an NCE for normal participants due to automatic motor inhibition, such that responses to targets following compatible primes are typically 10–30 ms *slower* than responses to targets following incompatible primes, (e.g., Eimer and Schlaghecken [2003]). Control participants all showed this expected NCE. The mean effect was −22.1 ms, with a range of −9 to −43 ms (Figure 6A). Overall mean RT ranged between 422 and 545 ms (and was not correlated with the size of the NCE). Mean error rate also showed an NCE, being 2.8% on compatible trials and 1.6% on incompatible trials (range 0.7%–7.5% across participants). The pre-SMA lesion control participant AG produced entirely normal results, with an NCE of −40 ms (Figure 6A; mean RT = 461 ms; error rates = 2.5% and 1.3% for compatible and incompatible trials, respectively). The results for lateral premotor lesion controls RS and VC, who also showed normal NCEs, are reported below.

Our hypothesis predicts that CB's SMA lesion should not only reduce or eliminate the NCE, but may lead instead to a *positive* compatibility effect or facilitation. According to our proposal, the critical role of the SMA is in automatic inhibition, but it may not be needed for the initial prime-related activation (which presumably occurs earlier in the processing stream). Thus, without the SMA, the inhibition would no longer exist, but the prime-related activation might still occur, leading to faster responses when target arrows followed compatible primes. CB's results conformed to these predictions, with a highly significant positive compatibility effect of 54 ms (t = 5.9, df = 381, p < 0.001). This result is not only a long way outside the normal range; it is entirely the reverse effect, indexing facilitation rather than inhibition (Figure 6A). CB's mean RT (533 ms) was within the normal range, as were his error rates (3.5% and 3% on compatible and incompatible trials, respectively). There was no significant difference between RT for left and right responses (539 versus 528 ms), and neither was there any significant asymmetry in the compatibility effect for left and right responses (63 versus 44 ms). Error rates were also similar for left and right (3.5% versus 3%).

If the inhibitory mechanism is effector specific (Eimer and Schlaghecken, 2003; Eimer et al., 2002), JR's smaller lesion of the SEF should affect the NCE for eye movements but spare it for hand movements if his SMA is largely spared. Consistent with this prediction, JR showed a highly significant NCE of −34 ms (Figure 6A; t = 3.9, df = 154, p < 0.001), with error rates of only 2.5% and 1.3% for compatible and incompatible trials. Thus, JR showed a normal priming effect for manual responses. Left responses were significantly faster than right responses (427 versus 467 ms; t = 4.7, df = 154, p < 0.001) and JR also made fewer errors for left responses (1.3% versus 3.8%), but there was no significant asymmetry in the compatibility effect for left and right responses (−23 versus −45 ms; interaction F(1,152) = 2.0; p = 0.16).

### Automatic Inhibition of Saccades

We extended the masked-prime paradigm into the eye-movement domain so that participants made leftward and rightward saccades, rather than button presses, in response to the target arrows. All other aspects of the procedure were identical to the manual condition. Control participants were predicted to show NCEs similar to those in Figure 6A, due to automatic inhibition of partially initiated saccade plans (Eimer and Schlaghecken, 2001). If this inhibitory process is mediated by the SEF, it should be reduced or absent in both CB and JR. If prime-related response activation still occurs in the absence of inhibition, we would predict positive compatibility effects for both these patients.

As expected, every control participant showed an NCE, ranging from −6.4 to −27.0 ms (mean = −19.6 ms; Figure 6B). This confirms that the saccadic task indexes automatic response inhibition in an analogous way to the manual task. Mean saccadic latency for each control participant ranged from 290–463 ms (and was not correlated with the size of the NCE), while error rates ranged from 1.3%–13% (group mean, 8% for compatible and 3.9% for incompatible trials). The lesion control participant, AG, also produced an NCE (−16 ms; see Figure 6B), with normal mean latency and error rate (381 ms and 9%).

CB's results displayed a pattern opposite to normal, with a significant *positive* compatibility effect of 13.6 ms (t = 2.5, df = 384, p = 0.01). Thus, as predicted, CB showed facilitation rather than inhibition on this eye movement task (Figure 6B), as well as on the previous hand-movement task. His mean latency (445 ms) was within the normal range, as were his error rates (3% on both compatible and incompatible trials). There was no significant difference between latencies for leftward and rightward saccades (450 versus 440 ms), and neither was there any significant difference in the compatibility effect for leftward and rightward saccades (15.1 versus 12.1 ms).

JR's results also displayed a significant positive compatibility effect of 16.4 ms (t = 3.6, df = 533, p < 0.001; Figure 6B). Error rates were 6% on compatible trials, and 9% on incompatible trials. Thus, although JR showed normal results for manual responses, his results were opposite to normal for saccades—a dissociation that mirrors the greater involvement of SEF than SMA in his lesion and confirms the effector-specific nature of the inhibitory mechanism. There was no overall difference between latencies for leftward and rightward saccades (359 versus 358 ms), but there was an asymmetry in the compatibility effect (26.2 versus 6.6 ms; interaction F(1,531) = 5.7. p = 0.018). This pattern of results was also reflected in the error rates, which were 7.7% overall for both left and right saccades, with an asymmetric compatibility effect (7.3% for left; −0.7% for right).

### Delayed or Absent Inhibition for Hand Movements?

Above, we reported a striking reversal of the manual compatibility effect for CB, as predicted if the SMA is responsible for automatic inhibition of manual response initiation. However, these results leave open the possibility that inhibition may be delayed rather than being altogether absent. That is, we have shown only that inhibition is absent at a prime-target interval of 150 ms. Now, we extend this to examine longer intervals, testing whether inhibition develops late in CB, or whether it is absent altogether, and we also increased the duration of the prime in case a stronger signal was needed to trigger the inhibitory mechanism in CB. First, we tested aged-matched control subjects over a range of onset asynchronies (SOAs), from 150–500 ms (see Experimental Procedures), in order to measure how long the inhibition normally lasts. We also examined AG and JR, to test whether their results remained normal over this range. Lastly, as further controls, we measured compatibility effects for patients RS and VC, who have extensive damage to lateral premotor areas.

**Figure 7:**
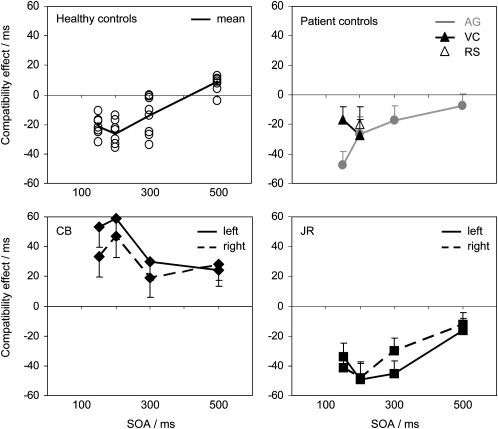
Results for Manual Responses with Varying Prime-Target Intervals The target arrows occurred 150, 200, 300, or 500 ms after the onset of the mask (the SOA). Results for healthy controls are individually plotted in the first panel (open circles), and their mean is shown as a black line. Results for AG, VC, and RS, the lesion control patients, are plotted in the top right panel (note that patients VC and RS did not complete all conditions, but showed NCEs nevertheless). Results for CB are shown in the bottom left panel, with right and left responses plotted separately. The compatibility effect remains reliably positive for both left and right at all SOAs, showing no sign of any inhibitory process. The last panel shows JR's results, which indicate normal inhibition. Error bars are the standard error of the difference between compatible and incompatible trials. See text for more details.

At SOAs of 150 and 200 ms, every healthy control participant showed NCEs, ranging between −11 ms and −36 ms (see Figure 7, top left panel). At 300 ms, most participants still showed a negative effect, while at 500 ms there was no longer any effect; i.e., responses for compatible trials were not longer than for incompatible trials. Mean RT for each participant ranged from 423–609 ms. Error rates ranged between 1% and 7% and showed slight NCEs at each SOA (group means, −1.7%, −1.6%, −0.8%, −2.3%). The pre-SMA lesion control participant, AG, produced a similar pattern of results, with NCEs of −48 ms (t = 5.0, p < 0.01), −27 ms (t = 2.9, p < 0.01), −17 ms (t = 1.7, p = 0.08) and −8 ms (t = 0.9), respectively, for SOAs of 150, 200, 300, and 500 ms (Figure 7, top right panel). Her mean RT was 443 ms, and error rate was 2%. Lateral premotor cortex patient VC also produced entirely normal results, with NCEs of −17 (t = 2.0, p < 0.05) and −27 ms (t = 3.3, p < 0.01) for SOAs of 150 and 200 ms, respectively (SOAs of 300 and 500 ms were not tested). Mean RT was 475 ms and error rate was 9.1%. Patient RS had difficulty with the button responses required by the task, and we therefore concentrated on an adapted version of one condition (SOA 200 ms; see Experimental Procedures). He showed relatively long RTs (mean 639 ms) and made many errors (21%), but despite this, we found an NCE within the normal range (−21 ms; t = 1.8, p = 0.07; 95% confidence interval −44 to +2 ms).

CB produced reliable *positive* compatibility effects at every SOA (Figure 7, bottom left panel). These were 43 ms (t = 3.1, p < 0.01), 53 ms, (t = 3.7, p < 0.001), 25 ms (t = 2.0, p < 0.05), and 26 ms, (t = 2.4, p < 0.05) for SOAs of 150, 200, 300, and 500 ms, respectively. This pattern clearly remains outside the normal range for all SOAs tested, and there is no sign that a negative phase develops at all. Mean RT was normal (524, 493, 492, 480 ms for each SOA). CB's overall error rates were also normal, but unlike controls, they showed positive compatibility effects (1.3 versus 8.8% for compatible versus incompatible at 150 ms SOA; 0 versus 2.5% for 200 ms SOA; 1.3 versus 2.5% for 300 ms SOA; 0 versus 1.3% for 500 ms SOA). There was no reliable difference between left and right responses or left and right compatibility effects.

JR's results also replicated and extended those of the initial experiment. He displayed clear NCEs of −37 ms (t = 3.8, p < 0.001), −48 ms (t = 4.5, p < 0.001), and −37 ms (t = 4.4, p < 0.001) at SOAs of 150, 200, and 300 ms, respectively (Figure 7, bottom right panel). Like controls, the effect had dissipated at an SOA of 500 ms. JR's left responses were faster than right responses (427 versus 449 ms), which may possibly indicate some damage to the left SMA, but note that some control participants showed equally large asymmetry in left and right response times. JR showed no asymmetry in the NCE.

### Delayed or Absent Inhibition for Saccades?

Lastly, we tested saccadic responses with varying intervals between prime and target. Above, we reported an unusual dissociation between manual and saccadic responses for JR. When tested with manual responses, there was a normal NCE. For saccadic responses, there was a reverse effect, like that in CB. We then replicated JR's normal NCE for manual responses at several SOAs. Now, we aimed to replicate the reversed effect for saccades and to test longer SOAs to discover whether inhibition is delayed or entirely absent.

**Figure 8:**
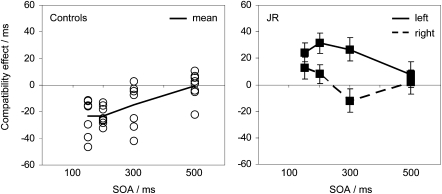
Results for Saccadic Responses with Varying Prime-Target Intervals Left panel: individual (open circles) and mean (black line) results for healthy controls, who all show NCEs for SOAs shorter than 300 ms. Second panel: Results for right and left responses from JR (error bars are the standard error of the compatibility effects for left and right responses). The compatibility effect for right responses is not reliably positive, but for SOAs of 150 and 200 ms it is clearly outside the normal negative range showed by controls. For left responses, the compatibility effect remains reliably positive until an SOA of 500 ms. See text for more details.

Results from controls mirrored those of the manual version of the task. At SOAs of 150 and 200 ms, every control participant showed NCEs ranging between −11 ms and −46 ms. At 300 ms, most participants had a reduced negative effect, and by 500 ms there was no longer any overall effect (see Figure 8, left panel). For the shorter SOAs, the negative effect was present for both leftward and rightward saccades in every subject. Mean latency for each subject ranged between 329 and 397 ms. Errors rates ranged between 0.5% and 12% and showed negative compatibility effects at all SOAs (groups means, −6%, −4.4%, −2%, −2.5%).

JR's results were clearly opposite to those of controls at SOAs of 150 ms and 200 ms (Figure 8, right panel). There was no reliable sign of inhibition at any SOA. There was also an asymmetry in the compatibility effects for leftward and rightward saccades (ANOVA interaction F_(1,521)_ = 10.2, p < 0.05). For rightward saccades, any positive compatibility effect dissipated by 300 ms. For leftward saccades, the positive compatibility effect remained large and reliable for SOAs of 150, 200, and 300 ms (respectively, 24 ms, t = 3.0, p < 0.01; 31 ms, t = 3.9, p < 0.001; 26 ms, t = 2.8, p < 0.01). Mean latency was within normal range (369 and 380 ms for leftward and rightward saccades, respectively). Error rates were relatively low (2.5%) and showed a positive compatibility effect of around 2.5% at each SOA, without any consistent left-right asymmetry. Thus, these results replicated and extended the striking dissociation between manual and saccadic responses in JR.

## Discussion

Following functional localization of the small lesions in CB and JR to the SMA and SEF, we tested whether this damage affected manual and oculomotor negative compatibility effects (NCE) in the masked-prime task—a measure of unconscious *automatic* motor inhibition. While healthy control participants showed entirely normal NCEs, CB and JR demonstrated reversed effects consistent with the extent of their lesions. The disruption occurred in an effector-specific manner, such that damage to the SMA affected performance on the manual task (Figures 6A and 7), while damage to the SEF disrupted the oculomotor task (Figures 6B and 8). When the SMA and SEF were spared in control patients AG (pre-SMA lesion), VC, and RS (lateral premotor cortex damage), responses were normal despite the fact that their lesions are many times larger than the lesions in CB or JR (Figure 4). Clearly, brain damage per se—even if it is extensive—does not lead to disruption of the NCE. Critically, the deficits observed in CB and JR on these tasks were restricted to abnormalities in the NCE and cannot be accounted for by general response impairments, simple perceptual deficits, or any response-accuracy tradeoff. Overall, response time and error rate were within the normal range, while diminished perceptual processing of the subliminal primes does not explain the results, because the primes actually caused robust facilitation in these two individuals.

### Automatic Inhibition in SMA and SEF

The results of this study were thus consistent with our hypothesis that one of the roles of the SMA and SEF is to contribute *automatic* inhibition of primed actions. Although our hypothesis may appear paradoxical for a region associated with voluntary actions, we consider automatic inhibition to be an important component of flexible, volitional behavior. It is now evident that well established condition-action associations can cause *automatic* subconscious motor activation—priming—even when the observer has no intention of making the associated movement (e.g., Tucker and Ellis, 2004). Importantly, previous studies have linked activity within the SMA and SEF with condition-action associations, although their focus was on the volitional activation of such associations, rather than automatic suppression of unconsciously triggered movement plans (Boettiger and D'Esposito, 2005; Chen and Wise, 1995; Lu et al., 2002; Olson et al., 2000; Rushworth et al., 2004; Sakai et al., 1999; Wise and Murray, 2000). According to our hypothesis, partial activations of movement plans can indeed be useful to facilitate actions. However, if the associated movements are not intended to be immediately executed, automatic inhibitory mechanisms within the SMA and SEF halt their further progress.

Finding specific deficits in patients with small lesions such as the ones described here is essential for establishing causal relationships between brain areas and functions. Imaging or neurophysiological studies establish *associations* between behavior and neuronal activity, rather than the behavioral *consequences* of interfering with activity. But, on the other hand, human lesion studies are usually limited by the fact that brain damage invariably extends well beyond the boundaries of any particular cortical area. This has been the case in the few studies of other patients with SMA lesions (e.g., Gaymard et al. [1993], Heide et al. [1996]). Crucially for this study, CB's and JR's lesions are—to the best of our knowledge—the smallest ever studied involving the SMA or SEF and on a par with the granularity of cortical areas revealed by fMRI or neurophysiology. The remarkable size of these lesions, combined with their effects on a simple behavioral task, allows us to advance the argument that there might be a causal relationship between the SMA and SEF and automatic inhibition of effector-specific condition-action associations.

Such automatic inhibition would act to restore balance whenever established condition-action associations automatically cause partial activation of a response, as they are known to do (Grezes and Decety, 2002; Leuthold and Kopp, 1998; Neumann and Klotz, 1994; Tucker and Ellis, 2004). The inhibition may be triggered entirely within the motor system by an automatic loop resulting directly from any partial activation that is not supported, for example, by further perceptual evidence and the intention to carry out that action (Eimer and Schlaghecken, 2003; Schlaghecken et al., 2006). Alternatively, the inhibition may be triggered by subsequent stimuli—an emergency break to favor a response to new stimuli instead of the response partially activated by the initial stimulus (Jaskowski and Przekoracka-Krawczyk, 2005; Lleras and Enns, 2006; Sumner, 2007a; see Supplemental Data for further details). Either way, automatic inhibition is likely to occur during many types of internally generated behavior because volitional actions, by definition, allow choice between several alternative condition-action possibilities, each of which may cause automatic response activation (Passingham, 1993; Rushworth et al., 2004; Tanji, 1996). We suggest that some activity in the SMA and SEF may be a low level consequence of this potential for unconscious response activation, rather than necessarily representing volitional planning or conscious goals.

Previously, automatic motor inhibition has been associated with the basal ganglia rather than the medial frontal cortex. Reduced or variable NCEs have been reported in patients with Parkinson's or Huntington's disease (Aron et al., 2003; Seiss and Praamstra, 2004). However, a more recent study extending the tested time course, as we have done here, reported no difference in the inhibitory pattern between Parkinson's disease patients and controls (Seiss and Praamstra, 2006). An fMRI investigation with healthy subjects has associated the inhibitory effect with deactivation of the striatum and thalamus (Aron et al., 2003). Thus, while the basal ganglia do not appear to be the source of inhibition, they may be subject to it, and such findings may be related to interactions with the SMA via cortico-striato-thalamic connections (Aron et al., 2003).

Interestingly, the unilateral lesions in CB and JR appear to have disrupted automatic inhibition of both left and right responses, consistent with the known *bilateral* representation of saccades and hand movements in the SEF and SMA (Fujii et al., 2002; Tehovnik et al., 2000). CB showed no evidence for asymmetrical effects in any part of the study (e.g., Figure 7). JR did show a larger facilitatory effect for leftward saccades than rightward saccades (Figure 8), consistent with previous findings in this patient of bilateral effects that were worse to the left (Husain et al., 2003; Parton et al., 2007). Note, however, that this asymmetry in facilitation was not accompanied by any large asymmetry in overall latency, which is what asymmetrical disruption of the inhibitory process would predict, rather than an asymmetric compatibility effect (see Figure S2 for further explanation).

### Role of SMA and SEF in Volitional Tasks

Many previous studies have implicated the supplementary motor complex (SMA and SEF) in the voluntary control of movement (for reviews, see Passingham [1993], Tanji [1996], Tehovnik et al. [2000]). For example, the SMA is associated with “self-initiated” actions that are not supported by visual guidance. Thus, monkeys with lesions involving the SMA are impaired when they have to initiate actions themselves or perform sequential movements, but not when each action is cued by sensory signals (Mushiake et al., 1990; Passingham, 1993; Shima and Tanji, 1998; Thaler et al., 1995). Neuronal recording and functional imaging studies are consistent with this view (Deiber et al., 1999; Romo and Schultz, 1987; Tanji and Shima, 1994). Similarly, the SEF has been implicated in the control of self-paced eye movements, saccade sequences, and antisaccades (Amador et al., 2004; Gaymard et al., 1993; Isoda and Tanji, 2002; Lu et al., 2002; Schlag-Rey et al., 1997). Moreover, patient JR has been found to have clear deficits in voluntary eye movement control, such as the ability to switch from a previous saccade plan or between pro- and antisaccades (Husain et al., 2003; Parton et al., 2007). Taken together, these findings demonstrate convincingly that both the SMA and SEF play crucial roles in volitional tasks, although the exact mechanisms underlying these roles remain debated.

Can any of the activity shown in SMA and SEF during paradigms used to test voluntary action, or any of the deficits in these tasks following brain damage, be related to the kind of automatic inhibitory mechanisms we have studied here? We have argued that inhibition of response alternatives can, paradoxically, be both an intrinsic component of volition and also automatic, as the masked-prime paradigm shows (Schlaghecken and Eimer, 2006; Sumner, 2007a). We would predict automatic inhibition to be most important in tasks containing stimuli strongly or recently associated with actions that now need to be discarded. Stop paradigms, change of plan, or rule reversal tasks, for example, all associate a stimulus with a certain response but require that response to be halted or changed on occasions. JR has shown volitional deficits only in tasks like these, in which one oculomotor plan is put in direct competition with another or a learned condition-action association has to be discarded, but not in pure blocks of saccades of all the same type, or in *unspeeded* saccade sequences that emphasize spatial sequence memory rather than rapid online control of action (Husain et al., 2003; Parton et al., 2007).

Tasks involving arbitrary condition-action associations may also engage automatic inhibition of partially activated responses. For example, when an animal learns a new set of arbitrary stimulus-response rules, the correct condition-action associations must be activated, while competing partially activated responses must be simultaneously inhibited. Neurones within the SEF show learning dependent changes (Chen and Wise, 1995, 1996), which might in part be related to such inhibitory effects. Patient JR also shows deficits in learning new stimulus-response mappings for eye—but not hand—movements, consistent with this account (Parton et al., 2007).

Automatic inhibition of unwanted responses is likely to be important also when we need to make speeded motor sequences or switch between sequences. Such tasks require selection either between components within a sequence or between old and new sequences. There is now considerable evidence that the SMA and SEF are both involved in aspects of sequence production (e.g., Isoda and Tanji, 2002; Lu et al., 2002; Mushiake et al., 1990; Nakamura et al., 1999; Shima and Tanji, 1998). Some studies suggest that the pre-SMA may be more important for acquisition of sequences (Nakamura et al., 1999). It is possible that one crucial role of the SMA/SEF in the execution of sequences is to inhibit responses that form part of a sequence but are not required at this point in the sequence, i.e., partially primed actions that need to be inhibited until the correct point (condition) in the sequence.

Another potential role for the SMA/SEF in sequence control might be inhibition of previously learned condition-action associations in old sequences. Thus in a given sequence, when presented with two targets—one above and one to the right of fixation—an animal may have to select a rightward saccade. However, in a different sequence, when confronted by the same stimuli, the correct response might be to make an upward eye movement (Lu et al., 2002). Under these two circumstances, competing stimulus-response activations are likely to occur, and it is crucial that previous condition-action associations are now automatically inhibited.

These examples illustrate how some of the activity documented in the supplementary motor complex in relation to a range of voluntary tasks may be related to the automatic inhibition mechanism we have described here. But note that the focus of previous studies was very different and those experiments were not specifically designed to address suppression of unwanted responses. Moreover, we would not claim that all deficits following lesions of the SMA complex can be accounted for by loss of automatic inhibition. The important point is that any SMA/SEF involvement in automatic inhibition by no means contradicts previous studies showing these areas to be important for a range of volitional behaviors. However, the approach developed here of examining potential automatic mechanisms has the virtue of attempting to decompose “volition” into precise and low-level component parts, as well as mapping such mechanisms onto discrete brain regions.

### Role of Pre-SMA in Volitional Tasks

The pre-SMA has been associated with voluntary control in a range of tasks requiring subjects to generate or freely select movements, execute action sequences, change from a preexisting movement plan to another, learn new condition-action associations or switch between them (Boettiger and D'Esposito, 2005; Garavan et al., 2003; Isoda and Hikosaka, 2007; Isoda and Tanji, 2004; Lau et al., 2006; Matsuzaka and Tanji, 1996; Nachev et al., 2005; Nakamura et al., 2005; Picard and Strick, 1996; Rushworth et al., 2002, 2004; Schlag-Rey et al., 1997; Shima et al., 1996; Stuphorn and Schall, 2002; Thaler et al., 1995; Ullsperger and von Cramon, 2001). Consistent with these observations, patient AG, whose large lesion includes the pre-SMA but spares the SEF and SMA, has difficulty rapidly changing from one motor plan to another (Nachev et al., 2007). Although this deficit in changing plan appears similar to one reported for patient JR (Husain et al., 2003), for the masked-prime task AG's results were normal throughout the study for both eye and hand. Thus, her volitional deficit cannot be explained on the basis of a loss of the automatic inhibitory mechanisms described here. In fact, AG's performance suggests that the masked-prime paradigm may tap an important distinction between the roles of SMA/SEF and the pre-SMA.

While the masked-prime task is effector specific, for eye or limb, many neural responses recorded in macaque pre-SMA are “amodal”; they are not specific to an effector (Fujii et al., 2002; Isoda and Tanji, 2004; Picard and Strick, 1996). Similarly, functional imaging studies in humans reveal that pre-SMA activation may occur in situations of either oculomotor or manual response conflict (Garavan et al., 2003; Nachev et al., 2005; Rushworth et al., 2002; Ullsperger and von Cramon, 2001). Thus, the contribution of the pre-SMA may not be at the level of particular effector-specific plans but rather at the level of a more general amodal competition between action goals (Isoda and Tanji, 2004; Picard and Strick, 1996), or it may play a role in switching from automatic to controlled action, as some very recent findings have suggested (Isoda and Hikosaka, 2007). By contrast, the SMA and SEF may deal with more tightly-linked stimulus-response representations, with automatic inhibition generated at a specific motor command level for these relatively simple condition-action associations.

Although such a distinction between pre-SMA and SMA/SEF is attractive, and SMA and SEF have been treated in parallel in our study, this is likely to be a simplification. The SEF, which is situated between the SMA and pre-SMA, has been reported to display some connectivity and activity patterns resembling pre-SMA and therefore appears to be more than just the oculomotor equivalent of the SMA (e.g., Chen and Wise [1995], Fujii et al. [2002], Johansen-Berg et al. [2004], Tehovnik et al. [2000]). Given that pre-SMA is not mapped in an effector-specific manner like SMA, the SEF may be considered to incorporate oculomotor populations of neurons from both rostral SMA and caudal pre-SMA. However, such a supposition is far from established and will require further investigation. From the point of view of the findings reported here though, the SEF and SMA appear to be crucial for effector-specific automatic inhibition, while pre-SMA does not.

### Conclusions

In summary, while the SMA and SEF have previously been associated with a range of voluntary, or “internally driven,” actions, our findings suggest that small lesions of these areas cause disruption to automatic effector-specific motor inhibition. Such automatic mechanisms may explain some of the activity in the SMA and SEF during “volitional” tasks. Automatic mechanisms have traditionally been considered distinct from goal-directed volitional behavior (e.g., Schneider and Shiffrin [1977]), but we suggest that automatic response inhibition plays an intrinsic role in efficient voluntary actions by keeping unwanted response activations in check to allow alternative actions to occur.

## Experimental Procedures

### Participants

Clinical details of the patients are given in Supplemental Data. Ten right-handed neurologically normal volunteers participated in the functional imaging. For the behavioral experiments, nine neurologically normal control participants were tested in experiment 1 (5 male, 4 female, aged 56–74, mean 67), while eight neurologically normal control participants were tested in each of experiments 2, 3, and 4. In experiment 2 there were 4 male, 4 female, aged 56–74 (mean age 66). In experiment 3 there were 3 male, 5 female, aged 59–75 (mean age 68). In experiment 4 there were 3 male, 5 female, aged 59–75 (mean age 69).

### Masked-Prime Behavioral Study

#### Apparatus

Stimulus presentation was performed by a PC-controlled Cambridge Research Systems (CRS) ViSaGe directly connected to a 21” Sony GDM-F520 Trinitron monitor. Stimulus presentation was synchronized with the screen refresh rate of 100 Hz, and timings were controlled and measured by the CRS clock and thus not subject to the errors produced by normal PC operating systems. Manual responses were made with a CRS CB6 button box. Eye movements were measured using an Applied Science Laboratories (ASL) model 504 high speed remote infra-red eye-tracker with an ASL 5000 series controller, which samples eye position at 240 Hz (chin and head rest also by ASL). Both vertical and horizontal displacement was recorded.

#### Automatic Inhibition of Manual Responses

The task was simply to respond to leftward or rightward pointing arrows as quickly as possible by pressing a left or right button. The arrow stimuli, >> or <<, were 1 deg by 1.5 deg and displayed in the center of the screen for 100 ms (see Figure 1). Before each target arrow stimulus, there occurred a prime arrow and a mask. The prime was either identical to the target arrow stimulus (compatible), or it was the opposite arrow stimulus (incompatible). The mask was made up of 30 lines. Each one was a random length (between limits), randomly oriented and positioned so that the centers of the lines made a 6 × 5 grid covering the prime stimulus. The prime was displayed for two frames (20 ms), then after a blank frame (10 ms) the mask appeared for ten frames (100 ms). This mask was sufficient to render the prime stimulus invisible to the participants (they were not aware of its presence and were at chance discriminating its direction when informed about it and asked to guess following the experiment). The target followed the mask after five frames (50 ms). These timings are similar to those used previously (e.g., Aron et al. [2003]; Eimer and Schlaghecken [2003]), and note that we have adopted the convention used in previous studies that the SOA is measured from the start of the *mask* to the start of the target, i.e., 150 ms. The target was displayed for 100 ms. Following the response there was 1000 ms pause, and then a fixation cross was displayed for 500 ms to signal the beginning of the next trial. This was followed by a blank of 300 ms, and then the prime-mask-target sequence as described above.

There were four types of trial: (1) leftward target with compatible prime; (2) rightward compatible; (3) left target with incompatible (rightward) prime; (4) rightward incompatible. There was always an equal number of each trial type, randomly ordered. Control participants performed 160, 200, or 240 experimental trials with a rest every 40 trials. JR performed 160 trials; CB and AG performed 400 trials. The experimental trials were preceded by 40 practice trials. Reaction times (RT) below 100 ms or above 800 ms were considered anticipations or lapses, respectively, and deleted from the analysis. Error responses were also removed from the RT analysis.

#### Automatic Inhibition of Saccades

The procedure was identical to that for manual responses, except as explained below. Saccadic responses were made instead of button presses and gray circles were displayed 8 deg to the left and right of the central arrow stimuli to act as targets for the leftward and rightward saccades. These circles were displayed continuously from the onset of the fixation cross until 800 ms after the arrow targets. The criterion for saccade detection was a velocity of 60°s^−1^, and saccadic onset was defined by a velocity > 22°s^−1^. Eye-position traces were inspected for all trials to check that the custom Matlab routine had correctly located saccades, and trials were discarded in which fixation was not maintained preceding target presentation. Trials in which the initial saccade was in the wrong direction were scored as errors. Saccades with latencies between 100 and 800 ms were accepted as responses to the target arrows (for some control subjects, stable eye tracking proved difficult and trials had to be rejected where the onset of the saccade was not clear). Controls performed 160 trials each, AG performed 240 trials, CB performed 400 trials, and JR performed 600 trials.

#### Delayed or Absent Inhibition?

The procedure was identical to that described above, except as explained below. In separate blocks, the SOA was 150, 200, 300, or 500 ms. Note that following Eimer and Schlaghecken (e.g., Eimer and Schlaghecken [2003]), the SOA is measured from the start of the mask to the start of the target. The prime duration was 30 ms (instead of 20 ms), which was followed as before by a blank frame (10 ms) and then the mask (100 ms). The SOA was manipulated by changing the number of blank frames between mask offset and target onset. Most participants performed 8 blocks of 80 trials each, including 2 blocks of each SOA in a counterbalanced order. Exceptions were VC, who performed 8 blocks of 48 trials for SOA of 200 ms and 7 blocks of 48 trials for SOA of 150 ms, and RS who performed 15 blocks of 48 trials only for 200 ms SOA.

## Supplemental Data

Document S1. Two Figures, One Table, and Supplemental Experimental Procedures
