# Supplementary material for: Human Medial Frontal Cortex Mediates Unconscious Inhibition of Voluntary Action
Source: Neuron. 2007 Jun 7;54(5):697–711. doi: 10.1016/j.neuron.2007.05.016 (PMC1890004; doi:10.1016/j.neuron.2007.05.016)
Supplement: Document S1. Two Figures, One Table, and Supplemental Experimental Procedures [file mmc1.pdf]

Neuron, Volume *54*

Supplemental Data

Human Medial Frontal Cortex Mediates Unconscious Inhibition of Voluntary Action

Petroc Sumner, Parashkev Nachev, Peter Morris, Andrew M. Peters, Stephen R. Jackson, Christopher Kennard, and Masud Husain

The Negative Compatibility Effect in Masked Priming

The negative compatibility effect (NCE) – slower responses to targets following compatible primes than following incompatible primes – has been reported and investigated in many masked prime experiments (Aron et al., 2003; Eimer and Schlaghecken, 1998, 2001, 2002; Eimer et al., 2002; Klapp, 2005; Klapp and Hinkley, 2002; Praamstra and Seiss, 2005; Schlaghecken and Eimer, 2000, 2002; Schlaghecken et al., 2003; Seiss and Praamstra, 2004); (see Eimer and Schlaghecken, 2003, for a review). It has been explained by a detailed theory in which motor activation occurring in the absence of supporting perceptual evidence is self-inhibited (Eimer and Schlaghecken, 2003; Schlaghecken et al., 2006). Motor plans for competing responses are also held to be mutually inhibitory, so that initial activation of one plan inhibits all others, and subsequent inhibition of that plan then releases the others from suppression.

This theory has been challenged, and aspects of it remain controversial. Most importantly for our study, it was suggested that NCEs measured in early studies might result not from motor inhibition but from perceptual interactions between the particular primes and masks used in these studies, where the mask was composed of overlapping prime stimuli (Lleras and Enns, 2004; Verleger et al., 2004). However, while it is generally acknowledged that this criticism was correct for these early studies, it has been shown not to apply to masks that are not composed of the prime stimuli (Klapp, 2005; Schlaghecken and Eimer, 2006; Sumner, 2007, in press), which is why we employed masks composed of randomly generated lines arranged in a grid.

The main point of controversy has been whether the primes must be invisible for the NCE to occur – whether there is any causal relationship between the absence of awareness of the prime and motor inhibition (Eimer and Schlaghecken, 2002; Jaskowski, in press; Jaskowski and Przekoracka-Krawczyk, 2005; Lleras and Enns, 2005, 2006; Sumner, 2007). In fact, NCEs have been measured with visible primes, so it appears that lack of perceptual support for motor activation is not a prerequisite for subsequent automatic inhibition (e.g. Jaskowski, in press; Lleras and Enns, 2006; Sumner et al., 2006). However, this debate is tangential to our purpose of using invisible primes to ensure that any NCE must be generated automatically – if the participant in unaware of the prime, he cannot volitionally suppress it. Related to the role of perceptual awareness is the question of whether the inhibition is truly "self-generated" within the motor system, or whether it is triggered by stimuli that follow the prime (e.g. the mask) – a "whoops response" or "emergency break" to halt activation of the response activated by the first stimulus and allow responses associated with new stimuli (Jaskowski, in press; Jaskowski and Przekoracka-Krawczyk, 2005; Lleras and Enns, 2006). While this debate is also tangential to our main purpose of simply studying whether SEF and SMA are associated with automatic inhibition (however it is triggered), the debate's resolution will have interesting implications for the exact roles of the SEF and SMA – how directly dependent on sensory input are these automatic sensory-motor mechanisms?

Functional Localisation of SEF and SMA in Healthy Participants

Although the *average* location of the supplementary motor complex, which includes SEF and SMA, across a group of subjects is closely predictable from the VCA line (Picard and Strick, 1996, 2001; Zilles et al., 1996), there is considerable variation from one subject to another (Curtis and D'Esposito, 2003; Grosbras et al., 1999; Picard and Strick, 1996). This variation is of the same order as the size of the functional regions, and is not correlated with sulcal landmarks or any other parameter easily identified with conventional MRI (Behrens et al., 2006; Mayka et al., 2006). Consequently, the only reliable way of localizing the SEF or SMA in a *specific subject* is to obtain a sequence of functional MRI images during the performance of a task known to engage these areas.

To localise lesions, we cannot rely on presence or absence of activity around the lesion, because normally functioning areas adjacent to the lesion may appear to be silent owing to focal signal loss caused by the deposition of haemosiderin in the damaged tissue. Conversely, non-functioning tissue at the edge of lesion may appear to be active in the absence of any true signal due to task-correlated head movement. However, we can make use of two relatively invariant relationships. First, within the SMC there is a rostrocaudal arrangement of the SEF and SMA (Picard and Strick, 1996). Second, functionally homology is mirrored by

anatomical homology *interhemispherically*. Thus the location of the SEF or SMA in the unaffected hemisphere is a good guide of the location of its homologue in the contralateral hemisphere (Grosbras et al., 1999; Picard and Strick, 1996). In patients with damage to one hemisphere only (as is usually the case with vascular lesions and surgical resections) localizing the target functional area in the good hemisphere is the *best* available method of predicting the likely location of the target relative to the lesion.

Although these two relationships are implicit in a wide literature, here we set out to confirm (and quantify) them in a cohort of 10 healthy subjects, employing simple oculomotor and limb movement tasks known to activate the SEF and SMA, respectively (see Experimental Procedures below). Coordinates of peak activation for oculomotor and manual activity in the superior frontal gyrus of each subject are given in Table S1. The mean difference between the cluster maxima corresponding to the SEF and the SMA, in MNI coordinates, is as follows. SEF_x - SMA_x = -1 mm, (SE=0.86), SEF_y-SMA_y = 4.8 mm (SE=2.09), SEF_z-SMA_z = –5.5 mm (SE=3.36). The location of the SEF is therefore marginally rostral to the SMA – as previously shown – and is an excellent guide to its location. The dimension of maximal variability – the z plane – matters least in our case because both patients have lesions that extend maximally in that plane. For each subject, the SEF and SMA in each hemisphere are confluent with their homologues in the opposite hemisphere. Thus the separation between each pair of homologous areas is within the intrinsic resolution of the data, which is estimated by the mean smoothness of the data as calculated by SPM: $FWHM_x$ = 11.3 mm (se=0.39); $FWHM_y$ = 11.7 mm (se=0.42); $FWHM_z$ = 8.6 mm (se=0.12). Thus the location of a functional area in one hemisphere is an excellent guide to its location in the other.

Participants	SEFx	SEFy	SEFz	SMAx	SMAy	SMAz
1	2	2	20	5	-2	32
2	2	12	34	2	16	38
3	2	12	37	5	-5	28
4	-9	-9	38	-9	-12	38
5	12	2	44	9	-5	47
6	2	5	28	2	-9	31
7	2	-23	34	2	-23	31
8	-2	5	34	5	5	37
9	2	12	26	2	5	56
10	-5	-2	28	-5	-2	40
Mean	0.8	1.6	32.3	1.8	-3.2	37.8

Table S1. MNI Coordinates of Peak Activation within the Superior Frontal Gyrus from Oculomotor and Limb Movement Tasks

Oculomotor activity defines the SEF and limb-movement activity defines the SMA.

Experimental Procedures for Functional Imaging

Behavioural Task

For the healthy volunteers, the behavioural protocol consisted of an oculomotor task and a limb motor task, performed in near darkness. The oculomotor task involved 8 blocks of alternately making eye movements and resting with eyes open, cued by a synthetic auditory word delivered through headphones. During an initial practice session, participants were familiarised with the cues and instructed to either fixate (rest) or to make self-paced horizontal eye movements of approximately 5° in amplitude (since they were in darkness it was explained that consistency of amplitude was neither possible nor important). All eye movements were tracked using an infra-red video based eye tracker (ASL) sampling at 60 Hz. The limb motor task was similarly blocked and cued, and involved making self-paced right or left finger or foot movements, separately in 4 blocks each. For the patients, the protocol was the same except that there were 12 blocks in the saccade task and 12 in the limb task, which concentrated only on finger movements (self-paced sequential finger-thumb oppositions with both hands simultaneously versus rest).

Data Acquisition

With CB, all functional imaging was done in a Siemens Trio 3.0T scanner. The parameters of the sequence were: TR = 2000ms, TE = 30ms, 32 axial slices, resolution = 3mm isotropic. Block (and total run) length were double that for the other studies.

For JR, two sets of functional localizers were performed - at 1.5T and 7T. The low field functional images were acquired on a 1.5 T Magnetom Vision scanner (Siemens, Erlangen, Germany) with a standard head coil. The functional runs consisted of series of 125 $T2^*$-weighted echoplanar images (TR = 4330ms, TE = 60ms, 40 axial slices, resolution = 3.5 x 3.5 x 3.0mm, gap = 0.5mm). Block length was 10 volumes (43.3 seconds). The high field functional images were acquired on a Philips Intera 7.0T scanner (at the Sir Peter Mansfield Magnetic Resonance Centre, Nottingham, UK). The oculomotor functional run consisted of a series

of 125 T2*-weighted, field-echo, echoplanar images (FOV= 64.00ap 39.30fh 64.00rl, TR = 3000ms, TE =25ms, 12 axial slices, resolution = 1 x 1 x 3.0mm, gap = 0.5mm). Block length was 10 volumes (30 seconds). Since the field of view was limited, the slices were centred on the VCA line in the midline, as judged by a sagittal scout image. The manual functional run was identical except that the TR was 2000 ms, and the block duration was therefore commensurately shorter (20 seconds).

For AG and the healthy volunteers, all functional imaging was done in the same Siemens Vision 1.5T scanner. The imaging parameters were as for JR's low field session.

Analysis

The functional data were analysed separately for each subject and scanner, using SPM2 or SPM5 (http://www.fil.ion.ucl.ac.uk/spm/). The first five images of each run were removed to allow for magnetic saturation effects. The images were realigned, smoothed with a Gaussian kernel of 8 mm FWHM (1.5T or 3T patients scans), 7 mm FWHM (healthy volunteers) or 2x2x6 mm FWHM (7T scans), and high-pass filtered to remove low-frequency signal drifts. For the 1.5T and 3T studies, to test for task-related activations the data were entered into a blocked, voxel-wise, general linear model (GLM) which included regressors modelling the tasks (as box-car functions convolved with the canonical haemodynamics response function (HRF)), their temporal derivatives, and head motion effects. The head motion regressors consisted of a series including the realignment parameters and their quadratics, both synchronously with the acquisition and time-shifted by one TR so as to model spin-history effects. For the 7T study, owing to the difference in TR, separate models were created for the oculomotor and manual tasks; the models were otherwise identically constructed. Task-specific effects were specified by appropriately weighted linear contrasts and determined with the t statistic on voxel-by-voxel basis. A statistical threshold of $p<0.001$ uncorrected for multiple comparisons was used to identify clusters of activation within the unaffected medial frontal lobe. For the 1.5T and 3T studies, so as to determine the location of activation in relation to the lesion, the mean echoplanar image was co-registered to the structural scan (resampled to 0.5 mm isotropic using 4th degree spline interpolation) and the co-registration parameters were then applied to the T maps. The co-registration was satisfactory as judged by the close overlap between the lesion on the structural and mean echoplanar images. Analysis for healthy volunteers was almost identical, but employed SPM5. A Gaussian kernel of 7 mm was used.

Control Patient Details

Patient AG, the lesion control participant, initially presented aged 52 following two generalised seizures. Neurological examination was unremarkable. Clinical MR imaging revealed a right dorsomedial frontal lesion whose margins fMRI confirmed to be anterior and medial to hand primary motor cortex. A tumour (grade 2 oligoastrocytoma) was surgically completely removed in conjunction with intra-operative electrical stimulation, ensuring that none of the areas designated for resection could elicit a motor response. Immediately following the operation, the patient demonstrated motor neglect of the left upper limb which resolved spontaneously. The experiments described here were performed 3 years after surgery when the patient was entirely asymptomatic and there were no abnormal clinical signs. Follow-up MR imaging 4 years after diagnosis has failed to demonstrate any evidence of tumour recurrence (the structural sequence used in Fig. 4 for AG was similar to CB's except that it was acquired on a Siemens Vision 1.5T scanner). Thus, AG has an extensive right SFG resection and provides a key control subject for comparison to JR and CB.

Two other patients, this time with longstanding and extensive lesions involving lateral pre-motor cortex, also participated as control patients. VC is a 60 year-old man who presented six years ago with a right-hemisphere stroke associated with left-sided limb weakness, dense left-sided visual neglect and left tactile extinction. His visual neglect and weakness improved remarkably, so that he was able to walk unassisted and make functional use of the left arm. However, he showed evidence of motor neglect, often failing to use the left arm even though it was strong. He now has some residual mild loss of dexterity of fine finger movements, but was able to make responses on the manual masked-prime task using both left and right hands. MRI demonstrates a large infarct in the territory of the right middle cerebral artery, involving right pre-motor (inferior and middle frontal gyrus) and motor cortex, extending also to involve prefrontal and parietal cortex (Fig. 4). Patients RS is a 71 year-old man who presented with a right-hemisphere stroke ten years ago. At that time he had a dense left-sided limb weakness, left visual neglect with intact visual fields and left-sided tactile extinction. Although his neglect resolved and power improved in the leg, power in the left upper limb did not improve and he is still unable to make any movements with the fingers of his left hand. We therefore asked him to make button-presses using the right index finger (for left targets) and right middle finger (for right targets). Although this is not the manner in which other subjects performed the task, it allowed us to determine whether there is any evidence of alteration of the normal NCE in this patient. MRI shows a very large old infarct involving the right premotor cortex (inferior and middle frontal gyrus) and motor cortex, extending to prefrontal, posterior parietal and superior temporal regions.

Reciprobit Analyses of JR and CB's Reaction Times

Reaction time data are often treated as if they are drawn from a single Gaussian distribution and for most purposes that is not an unreasonable assumption. However, closer analysis often reveals two separable component distributions whose reciprocals are both normally distributed but differ substantially in width and

location. The majority of responses are accounted for by a main, slow component distribution which is narrower in width than the minor, fast component that accounts for the remainder (Carpenter and Williams, 1995). Under conditions of urgency, or when the target is predictable not only do both component populations shift to the left (shorter latency) but the proportion of responses belonging to the minor component is often increased (Reddi and Carpenter, 2000). Moreover, pathological conditions may affect the two components differently (Ali et al., 2006). This suggests that mean reaction times are determined by at least two separable processes that are differentially modulated by the behavioural context, with the minor component seemingly increasing in significance in circumstances of greater response automaticity.

It is therefore conceivable that the changes in the overall mean reaction time in our two patients can be explained by a change in the proportion of responses derived from the minor process. If so, then damage to the supplementary motor complex may not have any impact on the main process involved in voluntary action, but only on the relative suppression of the faster, more automatic, minor process. Here we present evidence that this is not the case.

Maximum likelihood fits of the main (steep dotted lines) and minor (shallow dotted lines) components were generated for the raw RT data from the conditions showing a large facilitatory effect in patients JR and CB (Reciprobit Toolbox v 1.0, http://www.shadlen.org/mike/software/carpenterTools/contents.m). The results are plotted below (Figure S1), with the median reaction times as estimated by the main process fits in coloured numbers. It is clear that the minor component accounts for a very small proportion of responses, and that the difference in median reaction times for the main components is therefore very close to that observed for the population as a whole. Indeed, the minor component was impossible to identify in CB.

Interestingly, further analysis of the distributions in JR shows that the differences between the main components in the two conditions are better accounted for by a swivelling of the fits around a common intercept on the abscissa at infinity than a parallel shift (p=0.0373). If decision making is modelled as a stochastic process triggered by the stimulus and rising from some baseline to a fixed threshold at a linear rate, then a change in rate would be expected to produce a parallel shift whereas a change in the baseline a “swivel”. An unconscious congruent prime therefore appears to change the threshold for a response rather than accelerating the underlying process. This is consistent with a failure in suppressing prime-stimulated neurones encoding the sensorimotor transformation. CB’s manual data, however, does not show a clear predilection for one model over the other (p=0.380).

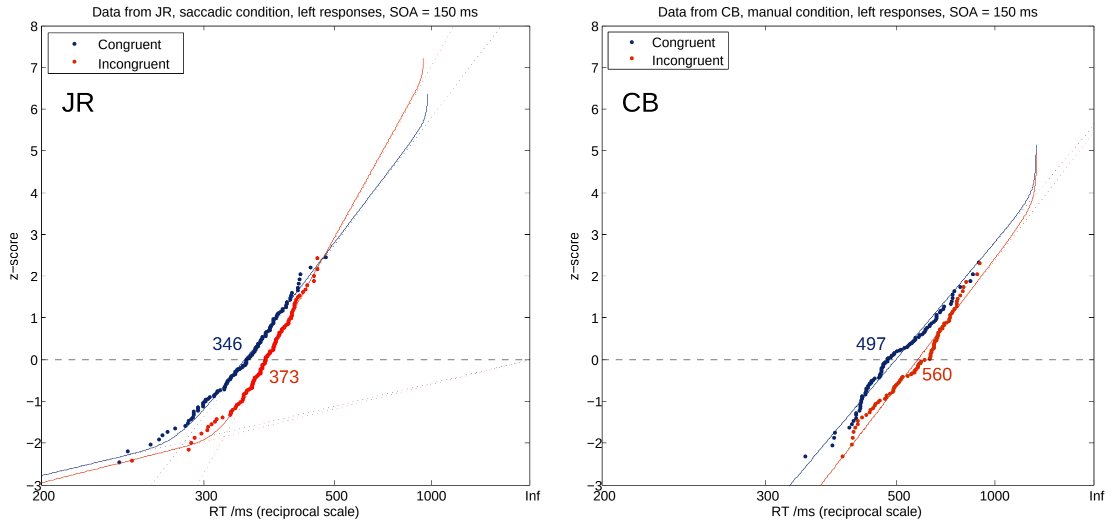

Figure S1. Reciprobit Analyses of JR and CB’s Reaction Times
See text for details.

Laterality

One important question regarding the function of SMA and SEF is the laterality of response effects. In CB's results both hands were equally affected (Figure 7), indicating that a lesion of the right SMA disrupts inhibition of both left and right response initiation. By contrast, JR's results showed a larger facilitatory effect for leftward saccades than rightward saccades (Figure 8), which might be taken to indicate asymmetrical disruption of the inhibitory process by his left SEF lesion. However careful consideration of the predicted results for asymmetrical inhibition leads to a different conclusion (Figure S2).

Consider a situation where inhibition occurs for rightward primes but not leftward primes. Under these circumstances, left compatible responses (i.e. left prime then left target) would be speeded because of uninhibited leftward prime activation. But left incompatible trials (i.e. right prime then left target) would also be speeded to some degree, this time due to rightward inhibition following the rightward prime. Given that the compatibility effect for left responses is calculated from RT for left incompatible trials minus RT for left compatible trials, and both of these are speeded (for different reasons), there would only be a small, if any, facilitatory effect for left responses. For right responses, on the other hand, compatible trials would be slowed due to inhibition following the rightward prime. However, incompatible trials (left prime then right target) would also be slowed, because of uninhibited activity from the leftward prime. Thus both responses would be slowed and there would be little compatibility effect, just as for left responses.

However, while asymmetric inhibition would create little asymmetry in the compatibility effect, it should create a marked asymmetry in overall latency. Left responses should all be facilitated while right responses should all be slowed. JR's results showed some evidence of such an asymmetry in latency RT, but it was not marked. Note however that this asymmetry occurred for both hand and eye responses, suggesting some asymmetrical disruption to manual inhibition associated with partial lesioning of the SMA as well as the SEF.

Thus while there may be some asymmetry in JR's inhibitory mechanisms, the asymmetry in the compatibility effect needs a different explanation. It is possible that disruption to the inhibitory process interacts asymmetrically with the response to the target, i.e. uninhibited incompatible priming causes greater interference for leftward saccades than for rightward saccades. Hence, greater difference between compatible and incompatible trials for leftward movements (Figure 8).

Overall, the more important conclusion is that the results from both CB and JR suggest that unilateral lesions to SMA and SEF disrupt inhibitory mechanisms bilaterally, for both leftward and rightward response initiation, consistent with the known bilateral representation of saccades and hand movements in the SEF and SMA (Fujii et al., 2002; Tehovnik et al., 2000).

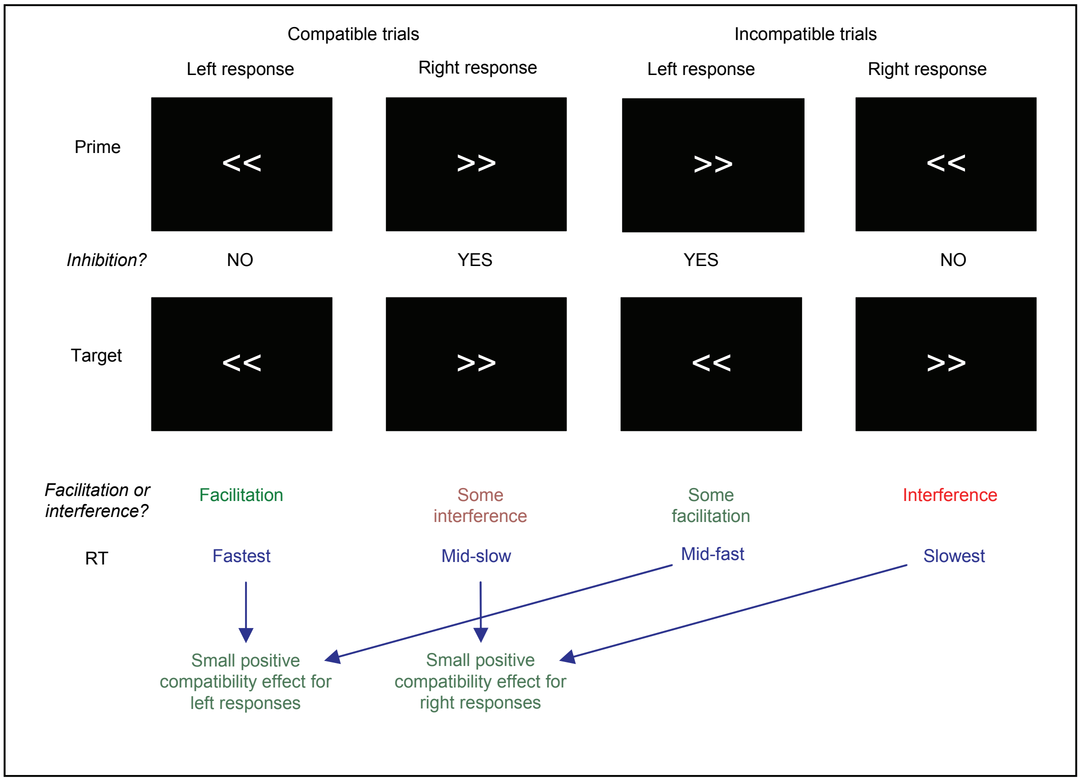

Figure S2. Predictions for Asymmetric Inhibition

If inhibition follows rightward, but not leftward primes, the outcome would be faster left than right response times, but counter-intuitively, no difference between compatibility effects for these responses.

Supplemental References

Ali, F.R., Michell, A.W., Barker, R.A., and Carpenter, R.H.S. (2006). The use of quantitative oculometry in the assessment of Huntington's disease. Exp. Brain Res. *169*, 237-245.
Aron, A.R., Schlaghecken, F., Fletcher, P.C., Bullmore, E.T., Eimer, M., Barker, R., Sahakian, B.J., and Robbins, T.W. (2003). Inhibition of subliminally primed responses is mediated by the caudate and thalamus: Evidence from functional MRI and Huntington's disease. Brain *126*, 713-723.
Behrens, T.E., Jenkinson, M., Robson, M.D., Smith, S.M., and Johansen-Berg, H. (2006). A consistent relationship between local white matter architecture and functional specialisation in medial frontal cortex. Neuroimage *30*, 220-227.
Carpenter, R.H.S., and Williams, M.L.L. (1995). Neural Computation of Log Likelihood in Control of Saccadic Eye-Movements. Nature *377*, 59-62.
Curtis, C.E., and D'Esposito, M. (2003). Success and failure suppressing reflexive behavior. J Cogn Neurosci *15*, 409-418.
Eimer, M., and Schlaghecken, F. (1998). Effects of masked stimuli on motor activation: Behavioral and electrophysiological evidence. J. Exp. Psychol.-Hum. Percept. Perform. *24*, 1737-1747.
Eimer, M., and Schlaghecken, F. (2001). Response facilitation and inhibition in manual, vocal, and oculomotor performance: Evidence for a modality-unspecific mechanism. J. Mot. Behav. *33*, 16-26.
Eimer, M., and Schlaghecken, F. (2002). Links between conscious awareness and response inhibition: Evidence from masked priming. Psychon. Bull. Rev. *9*, 514-520.
Eimer, M., and Schlaghecken, F. (2003). Response facilitation and inhibition in subliminal priming. Biol. Psychol. *64*, 7-26.
Eimer, M., Schubo, A., and Schlaghecken, F. (2002). Locus of inhibition in the masked priming of response alternatives. J. Mot. Behav. *34*, 3-10.
Fujii, N., Mushiake, H., and Tanji, J. (2002). Distribution of eye- and arm-movement-related neuronal activity in the SEF and in the SMA and Pre-SMA of monkeys. J. Neurophysiol. *87*, 2158-2166.
Grosbras, M.H., Lobel, E., Van de Moortele, P.F., LeBihan, D., and Berthoz, A. (1999). An anatomical landmark for the supplementary eye fields in human revealed with functional magnetic resonance imaging. Cereb. Cortex *9*, 705-711.
Jaskowski, P. (in press). The effect of nonmasking distractors on the priming of motor responses. Journal of Experimental Psychology - Human Perception and Performance.
Jaskowski, P., and Przekoracka-Krawczyk, A. (2005). On the role of mask structure in subliminal priming. Acta Neurobiol. Exp. *65*, 409-417.
Klapp, S.T. (2005). Two versions of the negative compatibility effect: Comment on Lleras and Enns (2004). Journal of Experimental Psychology-General *134*, 431-435.
Klapp, S.T., and Hinkley, L.B. (2002). The negative compatibility effect: Unconscious inhibition influences reaction time and response selection. Journal of Experimental Psychology-General *131*, 255-269.
Lleras, A., and Enns, J.T. (2004). Negative compatibility or object updating? A cautionary tale of mask-dependent priming. Journal of Experimental Psychology-General *133*, 475-493.
Lleras, A., and Enns, J.T. (2005). Updating a cautionary tale of masked priming: Reply to Klapp (2005). Journal of Experimental Psychology-General *134*, 436-440.
Lleras, A., and Enns, J.T. (2006). How much like a target can a mask be? Geometric, spatial, and temporal similarity in priming: A reply to Schlaghecken and Eimer (2006). Journal of Experimental Psychology-General *135*, 495-500.
Mayka, M.A., Corcos, D.M., Leurgans, S.E., and Vaillancourt, D.E. (2006). Three-dimensional locations and boundaries of motor and premotor cortices as defined by functional brain imaging: a meta-analysis. Neuroimage *31*, 1453-1474.
Picard, N., and Strick, P.L. (1996). Motor areas of the medial wall: a review of their location and functional activation. Cereb. Cortex *6*, 342-353.
Picard, N., and Strick, P.L. (2001). Imaging the premotor areas. Curr. Opin. Neurobiol. *11*, 663-672.
Praamstra, P., and Seiss, E. (2005). The neurophysiology of response competition: Motor cortex activation and inhibition following subliminal response priming. J. Cogn. Neurosci. *17*, 483-493.
Reddi, B.A.J., and Carpenter, R.H.S. (2000). The influence of urgency on decision time. Nature Neuroscience *3*, 827-830.
Schlaghecken, F., Bowman, H., and Eimer, M. (2006). Dissociating local and global levels of perceptuo-motor control in masked priming. J. Exp. Psychol.-Hum. Percept. Perform. *32*, 618-632.
Schlaghecken, F., and Eimer, M. (2000). A central-peripheral asymmetry in masked priming. Percept. Psychophys. *62*, 1367-1382.
Schlaghecken, F., and Eimer, M. (2002). Motor activation with and without inhibition: Evidence for a threshold mechanism in motor control. Percept. Psychophys. *64*, 148-162.
Schlaghecken, F., and Eimer, M. (2006). Active masks and active inhibition: A comment on Lleras and Enns (2004) and on Verleger, Jaskowski, Aydemir, van der Lubbe, and Groen (2004). Journal of Experimental Psychology-General *135*, 484-494.

Schlaghecken, F., Munchau, A., Bloem, B.R., Rothwell, J., and Eimer, M. (2003). Slow frequency repetitive transcranial magnetic stimulation affects reaction times, but not priming effects, in a masked prime task. Clin. Neurophysiol. *114*, 1272-1277.
Seiss, E., and Praamstra, P. (2004). The basal ganglia and inhibitory mechanisms in response selection: Evidence from subliminal priming of motor responses in Parkinson's disease. Brain *127*, 330-339.
Sumner, P. (2007). Negative and positive masked-priming – implications for motor inhibition. Advances in Cognitive Psychology *3*, (in press; available in preprint at www.ac-psych.org).
Sumner, P. (in press). Mask-induced priming and the negative compatibility effect. Experimental Psychology.
Sumner, P., Tsai, P.-C., Yu, K., and Nachev, P. (2006). Attentional modulation of sensorimotor processes in the absence of perceptual awareness. Proceedings of the National Academy of Science USA *103*, 10520-10525.
Tehovnik, E.J., Sommer, M.A., Chou, I.H., Slocum, W.M., and Schiller, P.H. (2000). Eye fields in the frontal lobes of primates. Brain Res Rev *32*, 413-448.
Verleger, R., Jaskowski, P., Aydemir, A., van der Lubbe, R.H., and Groen, M. (2004). Qualitative differences between conscious and nonconscious processing? On inverse priming induced by masked arrows. J. Exp. Psychol. Gen. *133*, 494-515.
Zilles, K., Schlaug, G., Geyer, S., Luppino, G., Matelli, M., Qu, M., Schleicher, A., and Schormann, T. (1996). Anatomy and transmitter receptors of the supplementary motor areas in the human and nonhuman primate brain. Adv. Neurol. *70*, 29-43.
